# RESPONDERS TO A PHYSICAL ACTIVITY COACHING PROGRAM IN COPD

**DOI:** 10.1093/geroni/igz038.2276

**Published:** 2019-11-08

**Authors:** Huong Q Nguyen, Amy Liu, Janet Lee, Anny Xiang

**Affiliations:** Kaiser Permanente Southern California, Pasadena, California, United States

## Abstract

Rationale: Physical inactivity is associated with worse outcomes in COPD. There remains a critical gap regarding the real-world effectiveness of improving physical activity (PA) in large representative samples of older adults with COPD and its impact on hospitalizations. Methods: A pragmatic randomized trial was conducted to determine the effectiveness of a 12-month home-based physical activity coaching intervention (Walk On!, WO) compared to standard care (SC) in 2,707 patients at high risk for COPD exacerbations. The WO intervention included collaborative monitoring of steps, semi-automated step goal recommendations, individualized reinforcement, and peer/family support. This is a subgroup analysis of 321 patients who were randomized to WO and participated in the program, matched to SC patients based on their propensity scores (PS). Multivariate cox proportional hazards models were used to determine differences in all-cause hospitalizations in the 12-months following randomization. Results: WO patients with low PS (n=160) were matched to 888 low PS SC patients and 161 WO patients with high PS (n=161) were matched to 405 high PS SC patients. Characteristics of the cohort were: age: 72±10; 54% females; 74% Caucasian; FEV1% predicted: 61±23. WO-low PS patients had lower risk of all-cause hospitalizations compared to SC-low PS patients [HR:0.69, (95%CI, 0.50, 0.96), P=.03]. WO-high PS patients did not have significantly lower hospitalization risk compared to SC-high PS patients [HR:0.87, (95%CI, 0.64, 1.20), P=.40]. Conclusions: Patients with COPD who had a lower propensity to participate in the physical activity coaching intervention had lower hospitalization risks compared to those with a higher participation propensity.

